# Entropy generation in the Malpighian tubule and its comparison with the mammalian nephron

**DOI:** 10.1007/s00360-026-01696-7

**Published:** 2026-07-27

**Authors:** Pedro Goes Nogueira-de-Sá, José Eduardo Pereira Wilken Bicudo, José Guilherme Chaui-Berlinck

**Affiliations:** 1 Departamento de Fisiologia, Instituto de Biociências da Universidade de São Paulo, São Paulo, SP Brazil; 2 School of Earth, Atmospheric and Life Sciences, University of Wollongong, Wollongong, Australia

**Keywords:** Malpighian tubules, Entropy generation, Excretory system, Insects, Mammals, Thermodynamics

## Abstract

Water and electrolyte regulation is essential for organismal survival. In mammals, the nephron operates a futile cycle of ultrafiltration and reabsorption that accounts for most of the entropy generation of the kidney. In contrast, insect Malpighian tubules form urine primarily through electrolyte secretion. Here, we develop a thermodynamic model of Malpighian tubule function in which entropy generation is estimated from electrical dissipation, with cationic urinary flow treated as current and transepithelial voltage as the potential difference. The model is applied to fluid secretion in the Malpighian tubules of the yellow fever mosquito, the stick insect, and the fruit fly, and to fluid reabsorption in the rectum of the desert locust and the fruit fly. The criterion of efficacy is the ratio between entropy generation and water flow, representing the energy dissipated per unit urinary volume. The results are compared with estimates for humans, mice, and dairy cattle, spanning eight orders of magnitude in body mass. The excretory systems of insects are shown to be more economical than those of mammals. Nevertheless, the differences are remarkably small across all species examined, despite the enormous variation in body size. These results confirm the hypothesis, proposed in the 1980s, that Malpighian tubules operate with greater efficacy than mammalian nephrons and further suggest that fundamental thermodynamic constraints shape the efficiency of epithelial transport systems across widely divergent organisms.

## Introduction

The maintenance of an adequate internal biochemical milieu is a central component of homeostasis and is energetically demanding (Natochin 2019). In terrestrial animals, water may become a scarce and unpredictable resource in addition to the challenges of electrolyte regulation. Consequently, excretory organs must be capable of dealing with both excess and shortage of water and salts.

There is a wide variety of excretory organs among metazoans, such as body surface, accumulating cells, protonephridia, metanephridia, kidneys, Malpighian tubules and others (Natochin 2019). Among these, the mammalian kidney holds particular interest in scientific and medical fields, and it is characterized by the glomerular nephron as its functional unit. This unit is composed of the glomerulus and a serial sequence of tubules, all perfused by specialized blood vessels. Primary urine production involves pressure-driven ultrafiltration by the glomerulus and subsequent reabsorption, driven by ion pumps, along with secretion processes which take place in the tubules and collecting ducts.

The Malpighian tubules of insects, on the other hand, rely on a very different way of primary urine formation (Cintra-Socolowski et al. 2016). They are blind-ended structures, directly connected to the transition between the mid- and the hindgut, specialized in the transport of water, electrolytes, nitrogenous wastes, and toxins, driven by ion pumps. Therefore, while mammalian nephrons rely on the ultrafiltration process, Malpighian tubule’s function relies on active ion secretion initiated in its distal end (Beyenbach et al. 2010; Dow et al. 2021).

**Fig. 1:**
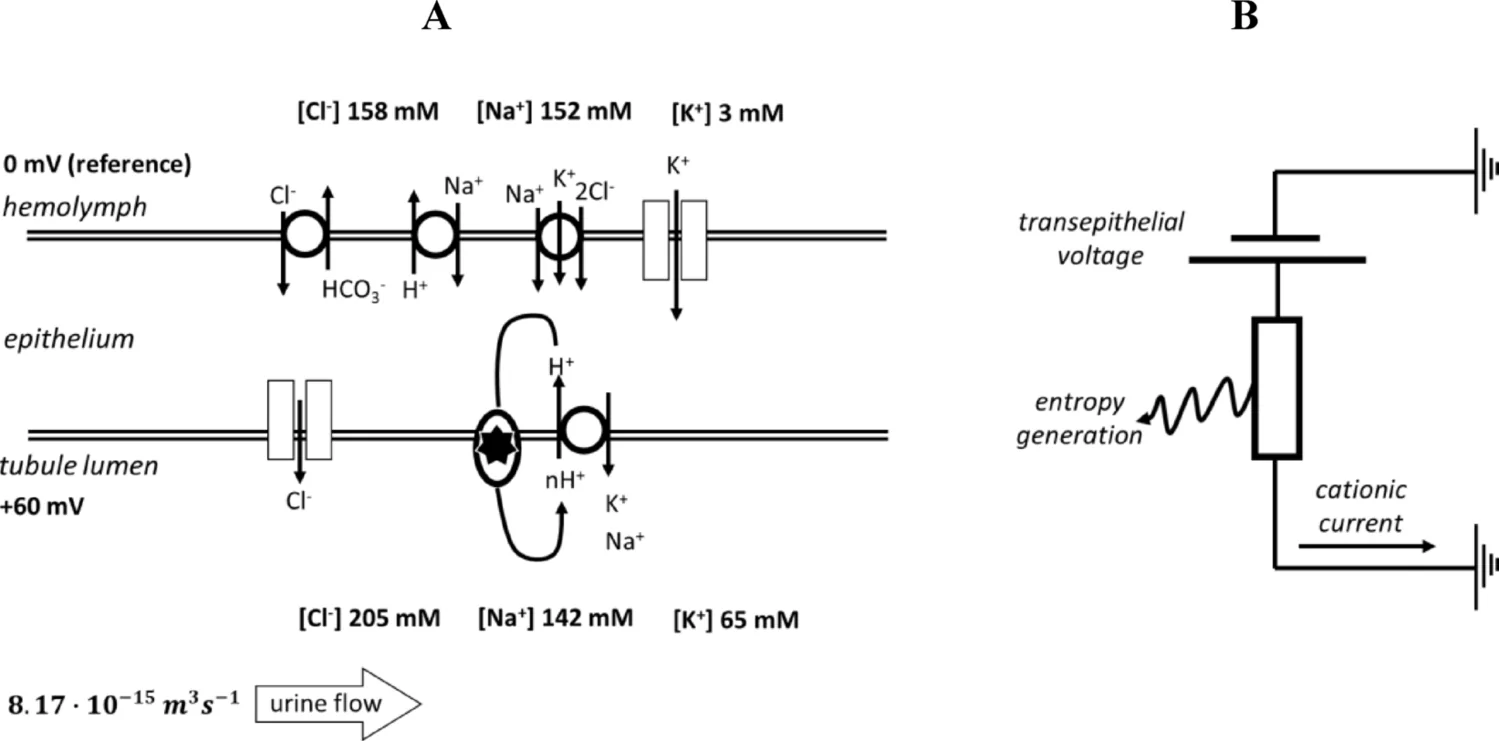
**A** Two-cell scheme of the secretion processes in the Malpighian tubule of *Aedes aegypti*. The key membrane channels (parallel rectangles) and exchangers (circles) are depicted. On the basolateral side of the epithelium (upper part of the figure), ions enter the epithelial cells passively via various cotransporters and antiporters. On the apical side (lower part of the figure), the active transport of sodium and potassium relies on a V-type H⁺-ATPase (black star), which generates a pH gradient across the membrane. This gradient is the driving force for the cation transport carried out by the NHA antiporter. Ion’s concentrations, voltage values, and water flow are indicated as well. Based on (Beyenbach et al. 2010). **B** Thermodynamic representation of a general Malpighian tubule in its secretory segment as an electrical circuit composed by a transepithelial battery that creates an electric current through a resistor generating entropy in the process

Most vertebrate epithelia depend on the nearly ubiquitous Na⁺/K⁺ ATPase in electrogenic cation transfer. Malpighian tubules depend either on V-type H⁺ ATPase or Na⁺/K⁺ ATPase, or even on both, to power transepithelial transport (Beyenbach 2003; Weng et al. 2003; Torrie et al. 2004; O’Donnell 2008; Beyenbach et al. 2010; Cintra-Socolowski et al. 2016; Halberg and Denholm 2024). The electrogenic proton or cation pump generates a local electrochemical potential that drives cation secretion responsible for sodium and, mainly, potassium secretion (see Fig. 1A). It is the active cation transport, similarly to the mammalian nephron reabsorption, that enables the movement of anions, water, and organic molecules through transcellular and paracellular pathways (Beyenbach 2003; Wu and Beyenbach 2003; Scott et al. 2004; Beyenbach et al. 2010). On the other hand, reabsorption seems to be primarily driven by Cl^−^ transport. Also, most of the secretion relies on a two-cell system for cation, anion and isotonic water transepithelial transfer (Dow et al. 2021; Halberg and Denholm 2024). It is important to note that from a thermodynamic standpoint the intricacies of ion pumps and cellular arrangements are not relevant (Fig. 1B).

As stated before, the homeostasis of the biochemical internal milieu is costly, and understanding the contribution of different organs to these processes is therefore essential. Given the biophysical and biochemical nature of excretion, thermodynamical modeling of transport phenomena is a valuable tool to deepen our knowledge of this facet of physiology. In this sense, it is possible to apply principles of irreversible thermodynamics to quantify the contribution of individual processes across the excretory organs (e.g. Lucia 2014a, b).

One can identify a thermodynamic futile cycle in the functioning of the nephron (Nogueira-de-Sá et al. 2025): fluid is filtrated and, subsequently, 99% reabsorbed. Thus, the work done on the reabsorbed fluid leads to dissipated power and entropy generation. In that study, the energy expenditure and entropy generation involved in urine formation in the mammalian kidney were estimated, and the model revealed that the pressure produced by active reabsorption in nephron tubules are approximately 750 times greater than the Starling forces. Moreover, such active transport accounts for more than 95% of total entropy generation in the glomerular nephron, including that associated with the total blood perfusion and glomerular ultrafiltration (Nogueira-de-Sá et al. 2025).

The present study focuses on estimating the entropy generation during the processes taking place in the Malpighian tubule, and comparing these results to those obtained for the mammalian nephron.

## Modelling

### The model for the Malpighian tubule

Since active ion transport involves the movement of electric charges across a membrane, the entropy generation of the epithelium was estimated using a model based on the dissipated power in an electric resistor.

The flows of sodium and potassium in the distal part of the Malpighian tubules are:

1a $$\:{\dot{V}}_{{\mathrm{N}\mathrm{a}}^{+}}\left(\mathrm{m}\mathrm{o}\mathrm{l}\:{\mathrm{s}}^{-1}\right)={\left[{\mathrm{N}\mathrm{a}}^{+}\right]}_{U}\:\cdot\:\:{J}_{{\mathrm{H}}_{2}\mathrm{O}}\:$$

1b $$\:{\dot{V}}_{{\mathrm{K}}^{+}}\left(\mathrm{m}\mathrm{o}\mathrm{l}\:{\mathrm{s}}^{-1}\right)={\left[{\mathrm{K}}^{+}\right]}_{U}\:\cdot\:\:{J}_{{\mathrm{H}}_{2}\mathrm{O}}$$

where [i^+^]_U_ is the urinary concentration of cation i^+^, and $\:{J}_{{\mathrm{H}}_{2}\mathrm{O}}$ is the urinary water flow. Units are indicated within parenthesis. The cationic electric current across the epithelium is estimated using ion flow:

2a $$\:{I}_{{\mathrm{N}\mathrm{a}}^{+}}\left({\rm A}\right)=F\:\cdot\:\:{\dot{V}}_{{\mathrm{N}\mathrm{a}}^{+}}$$

2b $$\:{I}_{{\mathrm{K}}^{+}}\left({\rm A}\right)=F\:\cdot\:\:{\dot{V}}_{{\mathrm{K}}^{+}}$$

2c $$\:{I}_{{Tot}^{+}}\left({\rm A}\right)={I}_{{\mathrm{N}\mathrm{a}}^{+}}+{I}_{{\mathrm{K}}^{+}}$$

where F is the Faraday’s constant (≅ 96,485.33 Coulombs per mol) and A is ampere.

The dissipated power, $\:{\dot{W}}_{{i}^{+}}$, i.e., the product of the thermodynamic force and the flux, in this case, electric potential difference, X, and current:

3 $$\:{\dot{W}}_{{i}^{+}}\left(\mathrm{w}\mathrm{a}\mathrm{t}\mathrm{t}\mathrm{s}\right)=X\:\cdot\:\:{I}_{{Tot}^{+}}$$

Notice that $\:{\dot{W}}_{{i}^{+}}$ focuses on the secretion phase of urine formation. If a near-complete water reabsorption in the hindgut would be considered, this value would approximately double. The mass-specific dissipated power and entropy generation are given by:

4a $$\:{\tilde{W}}_{{i}^{+}}\left(\mathrm{w}\mathrm{a}\mathrm{t}\mathrm{t}\mathrm{s}\:{\mathrm{k}\mathrm{g}}^{-1}\right)=\frac{{\dot{W}}_{{i}^{+}}}{{M}_{b}}\:$$

4b $$\:{\tilde{\upsigma}}_{{i}^{+}}\left(\mathrm{w}\mathrm{a}\mathrm{t}\mathrm{t}\mathrm{s}\:{\mathrm{K}}^{-1}\:{\mathrm{k}\mathrm{g}}^{-1}\right)=\frac{{\stackrel{\sim}{W}}_{{i}^{+}}}{T}$$

where the tilde indicates a “mass-specific” variable, M_b_ is body mass and T = 300 K is the absolute temperature in the surroundings (see “Discussion”).

For ion transport to occur, the V-type H+ type ATPase pumps protons by spending ATP at an efficiency estimated to be 90%. These protons are, then, exchanged back by the NHA membrane cation exchanger at an efficiency also estimated to be 90% (i.e., with minor leakage of protons). Since both pumps are at work during ion transport, the effective (eff) entropy generation is higher than the one given by Eq. (4b):

5 $${\tilde \sigma _{eff}}\left( {{\rm{watts~}}{{\rm{K}}^{ - 1}}{\rm{~k}}{{\rm{g}}^{ - 1}}} \right) = \frac{{{{{\rm{\tilde \sigma }}}_{{i^ + }}}}}{{0.9{\rm{~}}\cdot{\rm{~}}0.9}}{\rm{~}}$$

One additional relevant quantity is the mass-specific secretion, i.e., the urinary water flow per unit body mass:

6 $$\:\stackrel{\sim}{J}\:{(\:\mathrm{m}}^{3}{\mathrm{s}}^{-1}\:{\mathrm{k}\mathrm{g}}^{-1})=\frac{{J}_{{\mathrm{H}}_{2}\mathrm{O}}}{{M}_{b}}$$

Insects are a highly diverse Class and Malpighian tubule morphology, histology and number reflect such a diversity (Wigglesworth 1942). Also, experimental data from urine formation were obtained only in a few species. Thus, the present modelling was applied to three species where the available data allow for a quantitative approach. In the Results section we follow step-by-step the procedures of the modeling for *Aedes aegyptii*. Then, for *Carausius morosus* and *Drosophila melanogaster* we proceed directly to the results.

Datasets that allow assessment of the entropic budget associated with the reabsorptive phase of urine formation are scarce, and we were able to obtain data only for the fruit fly (*D. melanogaster*) and the locust (*Schistocerca gregaria*). Therefore, estimates for this phase are presented in a separate section.

### The model for the mammalian nephron

As presented elsewhere (Nogueira-de-Sá et al. 2025), the model for the mammalian nephron comprises the glomerular ultrafiltration and the subsequent isosmotic tubular water reabsorption. To account for a broader spectrum of analysis and a wide body mass span, we opted for humans [*Homo sapiens* (Nogueira-de-Sá et al. 2025)], mice [*Mus musculus* (Ito et al. 2017)] and dairy cattle [*Bos taurus* (Kume et al. 2008)] as our species of choice.

The urinary flow, J_U_, is the difference between the glomerular ultrafiltrate flow, J_g_, and the tubular reabsorption flow, J_reabs_:

7 $$\:{{J}_{U}\;\left({\mathrm{m}}^{3}{\:\mathrm{s}}^{-1}\right)={J}_{g}-\:J}_{reabs}$$

Daily urinary flow is 1% of glomerular filtration, while 90% of glomerular filtration is isosmotically reabsorbed by the tubules. The energy spent during active reabsorption is 14,975 J/mol NaCl (Deetjen and Kramer 1961; Greger 1996). Thus:

8 $$\:{J}_{reabs}\;\left({\mathrm{m}}^{3}{\:\mathrm{s}}^{-1}\right)=90\cdot\:\:{J}_{U}$$

9 $$\:{\mathrm{W}}_{reabs}\;\left(\mathrm{w}\mathrm{a}\mathrm{t}\mathrm{t}\mathrm{s}\right)=\mathrm{14,975}\:\cdot\:\:{\left[\mathrm{N}\mathrm{a}\mathrm{C}\mathrm{l}\right]}_{serum}\:\cdot\:\:{J}_{reabs}$$

The mass specific reabsorption rate is given by:

10 $$\:{\tilde{J}}_{reabs}\;\left({\mathrm{m}}^{3}{\:\mathrm{s}}^{-1}{\:\mathrm{k}\mathrm{g}}^{-1}\right)=\frac{{J}_{reabs}}{{M}_{b}}$$

Then, the dissipated power and the mass-specific entropy generation are given by:

11 $$\:{\stackrel{\sim}{\mathrm{W}}}_{reabs}\;\left(\mathrm{w}\mathrm{a}\mathrm{t}\mathrm{t}\mathrm{s}\:{\mathrm{k}\mathrm{g}}^{-1}\right)=\frac{{\mathrm{W}}_{reabs}}{{M}_{b}}$$

12 $$\:{\stackrel{\sim}{{\upsigma}}}_{reabs}\;\left(\mathrm{w}\mathrm{a}\mathrm{t}\mathrm{t}\mathrm{s}\:{\mathrm{K}}^{-1}\:{\mathrm{k}\mathrm{g}}^{-1}\right)=\frac{{\stackrel{\sim}{W}}_{\mathrm{r}\mathrm{e}\mathrm{a}\mathrm{b}\mathrm{s}}}{T}$$

## Results

### Input data summary

For the ease of reading we summarize data needed to obtain the quantitative results from the models of the Malpighian tubules and the nephrons in Table 1. The first line of the table contains body mass data arranged increasingly. Because the models are distinct in their inputs, part of the cells for mammals are left blank as well as part of the cells pertaining for the insects.

**Table 1:** Input data for the models

	*Drosophila*	*Aedes*	*Carausius*	*Mus*	*Homo*	*Bos*
M_b_ (kg)	1.0 · 10^− 6^	1.3 · 10^− 6^	8.2 · 10⁻⁴	2.0 · 10⁻²	7.0 · 10^1^	6.1 · 10^2^
${J}_{{\mathrm{H}}_{2}\mathrm{O}}$ (m^3^ s^− 1^)	1.83 · 10^− 14^	8.17 · 10⁻¹⁵	3.40 · 10⁻¹⁴	–	–	–
[Na^+^]_U_	35	142	5	–	–	–
[K^+^]_U_	128	65	145	–	–	–
X (mV)	55	60	30	–	–	–
${J}_{U}$ (m^3^ s^− 1^)	–	–	–	2.03 · 10⁻¹¹	2.00 · 10⁻⁸	1.76 · 10⁻⁷
${\left[{\rm NaCl}\right]}_{serum}$	–	–	–	150	140	145

Notice that concentrations are given in mol m^− 3^ (which has the same numerical value in mM) to obtain power in watts in Eq. (9)

### *Aedes aegypti*

*Aedes aegypti*, also known as the yellow fever mosquito, was chosen as the initial object of study due to more available data in the literature regarding its Malpighian tubules.

Flow of water in the lumen of Malpighian tubules of *Aedes aegypti* is approximately 0.49 nL per minute (Beyenbach et al. 2010). Therefore, the urine water flow, $\:{J}_{{H}_{2}O}$, is 8.17 · 10^–15^ m^3^ s^–1^.

Concentration of sodium in the lumen is 142 mM and of potassium is 65 mM (Fig. 1; Table 1). Therefore, according to equations < link rid="eqn2”>1</link> and 2:

13a $$\:{\dot{V}}_{{\mathrm{N}\mathrm{a}}^{+}}=142\:\cdot\:\:8.17\:\cdot\:\:{10}^{-15}=1.16\:\cdot\:\:{10}^{-12}\:\mathrm{m}\mathrm{o}\mathrm{l}\:{\mathrm{s}}^{-1}$$

13b $$\:{\dot{V}}_{{\mathrm{K}}^{+}}=65\:\cdot\:\:8.17\:\cdot\:\:{10}^{-15}=0.53\:\cdot\:\:{10}^{-12}\:\mathrm{m}\mathrm{o}\mathrm{l}\:{\mathrm{s}}^{-1}$$

13c $$\:{I}_{{\mathrm{N}\mathrm{a}}^{+}}=F\:\cdot\:\:1.16\:\cdot\:{\:10}^{-12}=1.12\:\cdot\:\:{10}^{-7}\mathrm{A}$$

13d $$\:{I}_{{\mathrm{K}}^{+}}=F\:\cdot\:\:0.53\:\cdot\:{\:10}^{-12}=0.51\:\cdot\:\:{10}^{-7}\mathrm{A}$$

13e $$\:{I}_{{Tot}^{+}}={I}_{{\mathrm{N}\mathrm{a}}^{+}}+{I}_{{\mathrm{K}}^{+}}=1.63\:\cdot\:\:{10}^{-7}\mathrm{A}$$

The electric potential across the epithelia is 60 mV, which combined with $\:{I}_{{Tot}^{+}}$ (Eq. 13e) gives the dissipated power, i.e., $\:{\dot{W}}_{{i}^{+}}$ (Eq. 3):

13f $$\:{\dot{W}}_{{i}^{+}}=60\:\cdot\:\:{10}^{-3}\:\cdot\:\:1.63\:\cdot\:\:{10}^{-7}=9.78\:\cdot\:\:{10}^{-9}\:\mathrm{w}\mathrm{a}\mathrm{t}\mathrm{t}\mathrm{s}$$

Thus, considering the mean mass of 1.3 milligrams for *Aedes aegypti* and an ambient temperature of 300 K, the mass-specific dissipated power and entropy generation are given by:

13g $${\tilde W_{{i^ + }}} = \frac{{{{\dot W}_{{i^ + }}}}}{{1.3{\rm{~}}\cdot{\rm{~}}{{10}^{ - 6}}}} = 7.52{\rm{~}} \cdot {\rm{~}}{10^{ - 3}}{\rm{~watts~k}}{{\rm{g}}^{ - 1}}$$

13h $$\:{\tilde{\upsigma}_{{i}^{+}}}=\frac{{\tilde{W}}_{{i}^{+}}}{300}=2.51\:\cdot\:\:{10}^{-5}\:\mathrm{w}\mathrm{a}\mathrm{t}\mathrm{t}\mathrm{s}\:{\mathrm{K}}^{-1}\:{\mathrm{k}\mathrm{g}}^{-1}$$

Then, from Eq. 5:

13i $${\tilde \sigma _{eff,{\rm{~}}Aedes}} = \frac{{{{{\rm{\tilde \sigma }}}_{{i^ + }}}}}{{0.9{\rm{~}}\cdot{\rm{~}}0.9}} = 3.10{\rm{~}}\cdot{\rm{~}}{10^{ - 5}}{\rm{~watts~}}{{\rm{K}}^{ - 1}}{\rm{~k}}{{\rm{g}}^{ - 1}}$$

Finally, from Eq. (6):

13j $$\:{\tilde{J}}_{Aedes}=6.28\:\cdot\:\:{10}^{-9}{\:\mathrm{m}}^{3}{\text{ s}}^{-1}\:{\mathrm{k}\mathrm{g}}^{-1}$$

### *Carausius morosus*

For *Carausius morosus*, a species of stick insect, we use experimental data from Ramsay (Ramsay 1955a, b):

Body mass: 0.82 g.

Water flow ($\:{J}_{{\mathrm{H}}_{2}\mathrm{O}}$): $\:3.40\:\cdot\:\:{10}^{-14}{\text{ m}}^{3}{\mathrm{s}}^{-1}$ (2.04 nL min^− 1^).

Luminal sodium concentration: 5 mM.

Luminal potassium concentration: 145 mM.

Transepithelial potential: 30 mV.

Therefore:

14a $$\:{\dot{V}}_{{\mathrm{N}\mathrm{a}}^{+}}=0.17\:\cdot\:\:{10}^{-12}\:\mathrm{m}\mathrm{o}\mathrm{l}\:{\rm s}^{-1}$$

14b $$\:{\dot{V}}_{{\mathrm{K}}^{+}}=4.93\:\cdot\:\:{10}^{-12}\:\mathrm{m}\mathrm{o}\mathrm{l}\:{\mathrm{s}}^{-1}$$

14c $${I_{To{t^ + }}} = F\cdot{\rm{~}}\left( {{{\dot V}_{{\rm{N}}{{\rm{a}}^ + }}} + {{\dot V}_{{{\rm{K}}^ + }}}} \right) = 4.92{\rm{~}} \cdot {\rm{~}}{10^{ - 7}}{\rm{~A}}$$

14d $$\begin{aligned} & {{\rm{\tilde \sigma }}_{{i^ + }}} = \frac{{30{\rm{~}} \cdot {\rm{~}}{{10}^{ - 3}}{\rm{~}} \cdot {\rm{~}}4.92{\rm{~}} \cdot {\rm{~}}{{10}^{ - 7}}}}{{0.82{\rm{~}}\cdot{\rm{~}}{{10}^{ - 3}}{\rm{~}}\cdot{\rm{~}}300}} \nonumber \\& \quad = 6.00{\rm{~}}\cdot{\rm{~}}{10^{ - 8}}{\rm{~watts~}}{{\rm{K}}^{ - 1}}{\rm{~k}}{{\rm{g}}^{ - 1}} \end{aligned}$$

14e $$\begin{aligned} & {\tilde \sigma _{eff,{\rm{~}}Carausius}} = \frac{{6.00{\rm{~}}\cdot{\rm{~}}{{10}^{ - 8}}}}{{0.9{\rm{~}}\cdot{\rm{~}}0.9}} \nonumber \\& \quad= 7.41{\rm{~}}\cdot{\rm{~}}{10^{ - 8}}{\rm{~watts~}}{{\rm{K}}^{ - 1}}{\rm{~k}}{{\rm{g}}^{ - 1}} \end{aligned}$$

14f $$\:{\tilde{J}}_{Carausius}=4.15\:\cdot\:\:{10}^{-11}{\:\mathrm{m}}^{3}{\text{ s}}^{-1}\:{\mathrm{k}\mathrm{g}}^{-1}$$

### *Drosophila melanogaster*

Experimental data for *Drosophila melanogaster* are from (O’Donnell and Maddrell 1995; O’Donnell et al. 1996):

Body mass: 0.001 g.

Water flow ($\:{J}_{{\mathrm{H}}_{2}\mathrm{O}}$): 1.83 · 10^− 14^ m^3^ s^− 1^ (1.1 nL min^− 1^) (O’Donnell and Maddrell 1995).

Luminal sodium concentration: 35 mM (O’Donnell et al. 1996).

Luminal potassium concentration: 128 mM (O’Donnell et al. 1996).

Transepithelial potential: 55 mV (measured at ambient temperature of 27 to 30 °C; values range from 44 to 68 mV) (O’Donnell et al. 1996).

Therefore:

15a $$\:{\dot{V}}_{{\mathrm{N}\mathrm{a}}^{+}}=0.64\:\cdot\:\:{10}^{-12}\:\mathrm{m}\mathrm{o}\mathrm{l}\:{\mathrm{s}}^{-1}$$

15b $$\:{\dot{V}}_{{\mathrm{K}}^{+}}=2.34\:\cdot\:\:{10}^{-12}\:\mathrm{m}\mathrm{o}\mathrm{l}\:{\mathrm{s}}^{-1}$$

15c $${I_{To{t^ + }}} = F\cdot{\rm{~}}\left( {{{\dot V}_{{\rm{N}}{{\rm{a}}^ + }}} + {{\dot V}_{{{\rm{K}}^ + }}}} \right) = 2.87{\rm{~}} \cdot {\rm{~}}{10^{ - 7}}{\rm{~A}}$$

15d $$ \begin{aligned} & {{\rm{\tilde \sigma }}_{{i^ + }}} = \frac{{55{\rm{~}} \cdot {\rm{~}}{{10}^{ - 3}} \cdot {\rm{~}}2.87{\rm{~}} \cdot {\rm{~}}{{10}^{ - 7}}}}{{1.00{\rm{~}}\cdot{\rm{~}}{{10}^{ - 6}}{\rm{~}}\cdot{\rm{~}}300}} \nonumber \\& \quad= 5.27{\rm{~}}\cdot{\rm{~}}{10^{ - 5}}{\rm{~watts~}}{{\rm{K}}^{ - 1}}{\rm{~k}}{{\rm{g}}^{ - 1}} \end{aligned} $$

15e $$ \begin{aligned} & {\tilde \sigma _{eff,{\rm{~}}Drosophila}} = \frac{{5.27{\rm{~}}\cdot{\rm{~}}{{10}^{ - 5}}}}{{0.9{\rm{~}}\cdot{\rm{~}}0.9}} \nonumber \\& \quad= 6.49{\rm{~}}\cdot{\rm{~}}{10^{ - 5}}{\rm{~watts~}}{{\rm{K}}^{ - 1}}{\rm{~k}}{{\rm{g}}^{ - 1}} \end{aligned} $$

15f $$\:{\tilde{J}}_{Drosophila}=1.83\:\cdot\:\:{10}^{-8}{\:\mathrm{m}}^{3}{\mathrm{s}}^{-1}\:{\mathrm{k}\mathrm{g}}^{-1}$$

#### Rectum/hindgut reabsorption

***Schistocerca gregaria***—data from Phillips et al. (1987).

For reabsorption in *S. gregaria*, the flow of Cl^−^ is the leading thermodynamic force. Since the absorbed liquid is released directly into the hemolymph, one cannot identify “the fluid being reabsorbed” per se. To overcome this limitation, we considered an isosmotic fluid reabsorption, as in the secretory initial phase of urine formation, and use the [Cl^−^] in the hemolymph to estimate the entropic cost of the process.

Body mass: 2.85 g

$$\:{J}_{{\mathrm{H}}_{2}\mathrm{O},\mathrm{r}\mathrm{e}\mathrm{a}\mathrm{b}\mathrm{s}}=4.72\:\cdot\:\:{10}^{-12}{\text{ m}}^{3}{\mathrm{s}}^{-1}\:(17\:{\upmu}\mathrm{L}\:\mathrm{h}^{-1})$$.

Hemolymph chloride concentration: 107 mM.

Transepithelial potential: 30 mV.

Therefore:

$$\:{\dot{V}}_{{\mathrm{C}\mathrm{l}}^{-}}=107\:\cdot\:\:4.72\:\cdot\:\:{10}^{-12}=5.05\:\cdot\:\:{10}^{-10}\:\mathrm{m}\mathrm{o}\mathrm{l}\:{\mathrm{s}}^{-1}$$

$${I_{{\rm{C}}{{\rm{l}}^ - }}} = F\cdot{\rm{~}}{\dot V_{{\rm{C}}{{\rm{l}}^ - }}} = 4.88{\rm{~}} \cdot {\rm{~}}{10^{ - 5}}{\rm{~A}}$$

$${{\rm{\tilde \sigma }}_{{i^ - }}} = \frac{{30{\rm{~}} \cdot {\rm{~}}{{10}^{ - 3}}{\rm{~}} \cdot {\rm{~}}4.88{\rm{~}} \cdot {\rm{~}}{{10}^{ - 5}}}}{{2.85{\rm{~}}\cdot{\rm{~}}{{10}^{ - 3}}{\rm{~}}\cdot{\rm{~}}300}} = 1.71{\rm{~}}\cdot{\rm{~}}{10^{ - 6}}{\rm{~watts~}}{{\rm{K}}^{ - 1}}{\rm{~k}}{{\rm{g}}^{ - 1}}$$

$$\begin{aligned} & {\tilde \sigma _{eff,{\rm{~}}Schistocerca~reabs}} = \frac{{1.71{\rm{~}}\cdot{\rm{~}}{{10}^{ - 6}}}}{{0.9{\rm{~}}\cdot{\rm{~}}0.9}} \nonumber \\& \quad = 2.11{\rm{~}}\cdot{\rm{~}}{10^{ - 6}}{\rm{~watts~}}{{\rm{K}}^{ - 1}}{\rm{~k}}{{\rm{g}}^{ - 1}} \end{aligned} $$

***Drosophila melanogaster***—data from Andersen and Overgaard (2020).

For *D. melanogaster* the computations are a little different because the study gives the cationic currents straightway. Therefore, there is no need of Eq. (1), and the total cation current ($\:{\dot{V}}_{{\mathrm{K}}^{+}}+{\dot{V}}_{{\mathrm{N}\mathrm{a}}^{+}}$) is 1.47 · 10^− 12^ mol s^− 1^.

$${I_{To{t^ + }}} = F\cdot1.14\cdot~{10^{ - 12}} = 1.42{\rm{~}} \cdot {\rm{~}}{10^{ - 7}}{\rm{~A}}$$

$$\:Transepithelial\:potential=30\:\mathrm{m}\mathrm{V}$$

$$\begin{aligned} & {\tilde \sigma _{eff,{\rm{~}}Drosophila~reabs}} = \frac{{1.42\cdot{{10}^{ - 7}}\cdot30 \cdot {{10}^{ - 3}}}}{{1.00\cdot{{10}^{ - 6}}\cdot300\cdot0.9\cdot0.9}} \nonumber \\& \quad = 1.75{\rm{~}}\cdot{\rm{~}}{10^{ - 5}}{\rm{~watts~}}{{\rm{K}}^{ - 1}}{\rm{~k}}{{\rm{g}}^{ - 1}} \end{aligned}$$

$${J_{{{\rm H}_2}{\rm O},reabs}} = 1.67\cdot{10^{ - 14}}\,{{\rm{m}}^3}{{\rm{s}}^{ - 1}}{\rm{~}}\left( {1.0{\rm{~nL~min}} - 1} \right)$$

#### Mammals

For mammals, the model presented in a previous section is extensively discussed elsewhere (Nogueira-de-Sá et al. 2025). Therefore, we give only the relevant results in the next section without a detailed step-by-step procedure as we did for the insects.

### Results summary

Table 2 summarizes the results for the model for insects’ Malpighian tubules (Eqs. 1 to 6) and for the mammalian nephrons (Eqs. 7 to 12). Table 3 summarizes the results for the rectum/hindgut reabsorption process for *D. melanogaster* and *S. gregaria*.

**Fig. 2:**
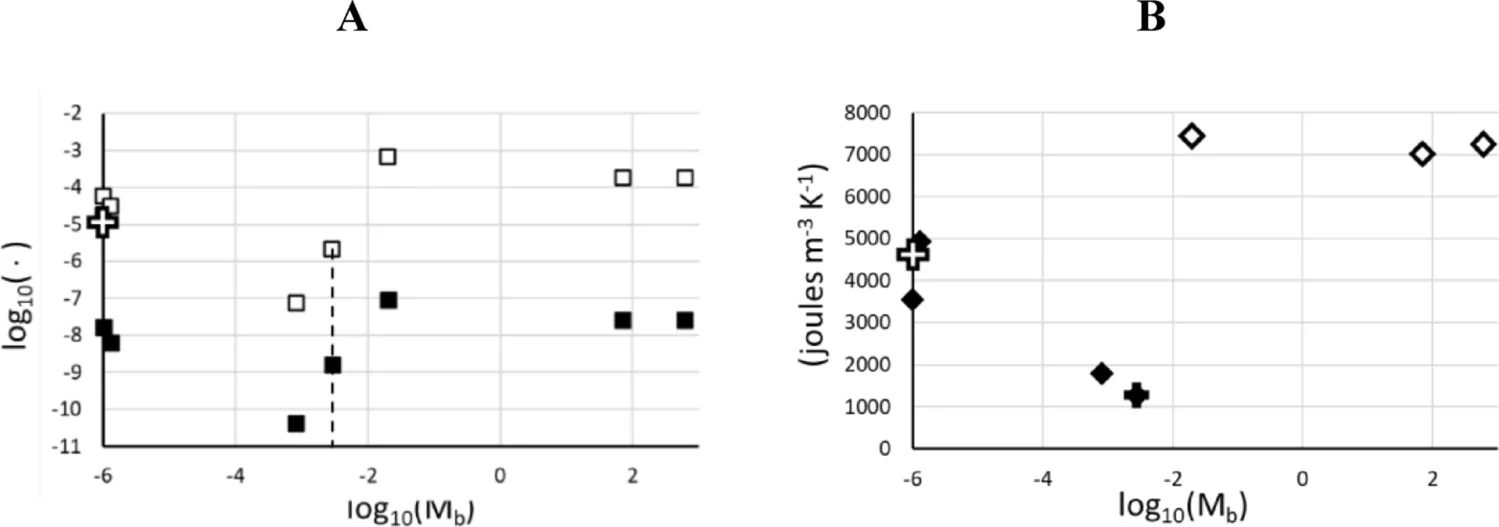
**A** Log–log plot of mass-specific water flow (closed squares ■) and of mass-specific entropy generation (open squares □) in relation to body mass. Dashed vertical line indicates the reabsorptive values for the locust *S. gregaria*. Open cross: reabsorptive $\:\tilde{\sigma}$ for *D. melanogaster*. Notice that the reabsorptive and the secretive mass-specific water flows for *D. melanogaster* are too similar to each other to be discerned in the plot. **B** Ratio between entropy generation and water flow rate ($\:\tilde{\sigma}/\tilde{J}$ last line of Tables 2 and 3) in relation to log_10_ of body mass. Open diamonds (◊): mammals. Closed diamonds (♦): insects (secretion). Open cross: secretive plus reabsorptive values for *D. melanogaster*. Closed cross: reabsorptive value for *S. gregaria*

Figure 2A shows the mass-specific water flow rate $\:\tilde{J}$ and mass-specific entropy generation $\:\tilde{\sigma}$ versus body mass in a log-log plot. Figure 2B shows $\:\tilde{\sigma}/\tilde{J}$ (watts m^− 3^ K^− 1^) versus log of body mass.

**Table 2:** Results for the models of insect and of mammalian excretory systems

	*Drosophila*	*Aedes*	*Carausius*	*Mus*	*Homo*	*Bos*
${\dot{W}}_{{i}^{+}}\:$ $\mathrm{o}\mathrm{r}\:{W}_{reabs}$	1.95 · 10^− 8^	1.21 · 10^− 8^	1.82 · 10^− 8^	4.11 · 10^− 3^	3.77 · 10^0^	3.44 · 10^+ 1^
${\tilde{W}}_{{i}^{+}}\:$ $\mathrm{o}\mathrm{r}\:{\tilde{W}}_{reabs}$	1.95 · 10^− 2^	9.29 · 10^− 3^	2.22 · 10^− 5^	2.06 · 10^− 1^	5.39 · 10^− 2^	5.61 · 10^− 2^
$\tilde{J}$	1.83 · 10^− 8^	6.28 · 10^− 9^	4.15 · 10^− 11^	9.15 · 10^− 8^	2.57 · 10^− 8^	2.58 · 10^− 8^
$\tilde{\sigma}$	6.50 · 10^− 5^	3.10 · 10^− 5^	7.41 · 10^− 8^	6.85 · 10^− 4^	1.80 · 10^− 4^	1.87 · 10^− 4^
$\tilde{\sigma}/\tilde{J}$	3549	4926	1786	7488	6988	7238

**Table 3:** Results for the model of rectum/hindgut reabsorption

	*D. melanogaster*	*S. gregaria*
${\dot{W}}_{reabs}$	4.25 · 10^− 9^	1.46 · 10^− 6^
${\tilde{W}}_{reabs}$	4.25 · 10^− 3^	5.13 · 10^− 4^
$\tilde{J}$	1.67 · 10^− 8^	1.66 · 10^− 9^
$\tilde{\sigma}$	1.75 · 10^− 5^	2.11 · 10^− 6^
$\tilde{\sigma}/\tilde{J}$	1048	1275

## Discussion

Excretory organs exert a constant role in keeping the internal milieu adjusted to external and internal conditions. In the case of terrestrial animals, this role goes beyond solute balances because water (i.e., organismal free water) balance must also be considered in the process due to the potential absence of this substance at a constant and/or predictable time-basis. In the present study we modeled and analyzed the energetics of urine formation in the excretory system of insects, the Malpighian tubules, and compared them to the mammalian nephrons.

### General aspects of the study

Entropy generation represents a loss of useful work. In the mammalian kidney, this loss is due to the thermodynamic futile cycle established between filtration and reabsorption (Nogueira-de-Sá et al. 2025). Here we show that, in the case of Malpighian tubules, entropy generation results from the electric charge flow (i.e., the equivalent to an electrical current) due to a certain voltage.

In this regard, we choose to compute entropy generation as occurring at an ambient temperature of 300 K for all the species. While this is obviously not true inter-species, intra-species and even throughout a single individual life, the range in terms of absolute temperature is very narrow, and, for instance, calculations using 285 K for some and 315 K for others would make little difference (approximately 5%). Therefore, 300 K was selected to isolate the effect of dissipated power on entropy generation. This approach ensures that the analysis remains focused on the physiological conditions of the Malpighian tubules and the nephrons rather than on surroundings degradation.

In a broad sense, while mammals are somewhat stereotyped in relative water reabsorption in the nephrons, i.e., only about 10% of the total ultrafiltered water is under central regulation (Koeppen and Stanton 2007; Chaui-Berlinck 2025), reabsorption in the proximal Malpighian tubules and rectum/hindgut is much more diverse in insects (Wigglesworth 1942; Phillips et al. 1987; Naseem et al. 2023). This intrinsic difference is probably due to the much higher diversity of insects in relation to mammals. The physiological and ecological diversity of these two groups is reflected in their respective numbers of species: while mammals comprise over 6000 species (Burgin et al. 2018), insects are estimated to include around 5 million species (Stork 2018). Consequently, greater phenotypic diversity among insects is expected, including differences in active transport properties, as estimated from our modeling.

At first glance, one would hypothesize that the energetics of the urine formation in Malpighian tubules would somehow reflect such a diversity. Our analysis shows that this is true when one focuses in particular cases. However, in a broad sense, this is not the case, not even in comparison with mammals.

### Comparisons among the insects

#### Secretion in *Aedes aegypti* and *Carausius morosus*

The mass-specific secretion and the entropy generation in stick insects were estimated to be 2 to 3 orders of magnitude lower than in yellow fever mosquitoes (Table 2, lines 3 and 4, Fig. 2A). These differences are influenced by various physiological and ecological factors, making it challenging to identify the primary contributors to variations in urinary production. Diet plays a crucial role in determining urine formation. Yellow fever mosquitoes primarily feed on fruits, with females also consuming blood to support egg maturation (e.g., Wigglesworth 1942; Tenywa et al. 2024). In contrast, stick insects are herbivorous animals that feed on fresh leaves (Blüthgen et al. 2006; Blüthgen and Metzner 2007). The solute and water content of these diets differ significantly, directly impacting urine composition and the thermodynamics of urinary processes. Additionally, since fruits contain high levels of sugar, yellow fever mosquitoes likely experience greater metabolic water production through glucose catabolism. This increased metabolic water production could contribute to the higher mass-specific secretion observed in their Malpighian tubules compared to stick insects.

#### Reabsorption in *Drosophila melanogaster* and *Schistocerca gregaria*

Regarding the reabsorption process, the comparison between the fruit fly and the locust (Table 3) is remarkably analogous to the one presented in the previous paragraph. Our results show that in spite of the latter having a body mass 2850 times the one of the former, the mass-specific flow and entropy generation of the locust are one-tenth of those of the fruit fly. As in the comparison made above between the yellow fever mosquito and the stick insect, these mass-specific low values of water flow and entropy generation probably reflect diverse habitats and food habits of *D. melanogaster* and *S. gregaria* in a very similar way as for the mosquitoes and the stick insects. Fruit flies have a much more predictable access to food rich in water and sugar in a humid environment. By its turn, the desert locust lives in more arid lands with less predictable access to food and water (e.g. (Despland and Simpson 2000).

In spite of the differences noted in the two preceding paragraphs, when one compares the entropic budget in relation to the water flow, i.e., $\:\tilde{\sigma}/\tilde{J}$, these differences almost vanish, which leads us to our next topic.

### Insects and mammals: important differences …

The $\:\tilde{\sigma}/\tilde{J}$ ratio gives the entropy generation per unit volume moved in the excretory system. Since for all the species we computed the entropy generation in regard to 300 K surroundings, the values can, for comparisons, be interpreted as the dissipated power to move one unit volume of water in the excretory systems.

Computations of the reabsorptive phase for the fruit fly and the desert locust reveal that this phase seems to account for the same energetic budget per unit volume as the secretory one. Therefore, even considering an almost complete fluid reabsorption, the $\:\tilde{\sigma}/\tilde{J}$ values are considerable below than those for mammals (Fig. 2B).

Simply put, the insect excretory system is more economical than that of mammals. More than 40 years ago, Maddrell hypothesized that this would be the case (Maddrell 1981). According to his hypothesis, Malpighian tubules generally operate at lower flow rates than mammalian nephrons because insects can tolerate much lower rates of nitrogenous waste elimination. The resulting low urinary flow allows useful compounds to be reabsorbed at correspondingly low rates, further decreasing the energetic burden of the excretory system (Maddrell 1981). Our results give strong support for this hypothesis.

Both mammals and insects evolutionarily transitioned to land where water is an unpredictable good. Why one group would operate at a lower energy demand in its excretory system than the other is an important and interesting unanswered question. A very probable reason lies in scaling. Even the smallest mammal has a body mass of at least 10 times the one of a large insect. This imposes a large area-to-volume ratio in the insects that might causes severe dehydration in very short periods of time (Maddrell 1981; O’Donnell 2008).

Interestingly, Maddrell claims that the tubules must have low permeability in order to allow for effective elimination of some and retention of other substances ((Maddrell 1981) - pg. 11). This is exactly what we have concluded regarding the differences in permeability between the glomerular capillaries and the reabsorptive tubules (Nogueira-de-Sá et al. 2025). And this leads us to our final topic.

### … and striking similarities

As we stated before, we can treat the $\:\tilde{\sigma}/\tilde{J}$ value directly as an energetic budget instead of an entropic one due to the way we computed it in this study. Then, in spite of eight orders of magnitude in body mass span, the values of this ratio are restricted to the same order magnitude (10^8.79^ variation in body mass, 10^0.62^ in $\:\tilde{\sigma}/\tilde{J}$). Therefore, while the morphology and functioning of the excretory organs are very different, their efficiencies are very similar.

Since active transport is the main source of entropy generation in mammalian kidneys (Nogueira-de-Sá et al. 2025), and it is also the primary process driving urine formation in insects, this similarity in entropy generation per unit of active transport likely reflects the conservation of fundamental features of membrane transport physiology. These results suggest that essential thermodynamic boundaries shape the efficiency of epithelial transport systems across widely divergent organisms. Therefore, biophysical and biochemical constraints governing membrane transport appear to be stronger than the evolutionary forces that have diverged mammal and insect lineages, and our findings are consistent with this interpretation.

## Data Availability

No datasets were generated or analysed during the current study.
